# One Health Core Competency Domains

**DOI:** 10.3389/fpubh.2016.00192

**Published:** 2016-09-13

**Authors:** Rebekah Frankson, William Hueston, Kira Christian, Debra Olson, Mary Lee, Linda Valeri, Raymond Hyatt, Joseph Annelli, Carol Rubin

**Affiliations:** ^1^Centers for Disease Control and Prevention, Atlanta, GA, USA; ^2^Oak Ridge Institute for Science and Education, Oak Ridge, TN, USA; ^3^University of Minnesota, Minneapolis, MN, USA; ^4^Chiang Mai University, Chiang Mai, Thailand; ^5^Tufts University, Boston, MA, USA; ^6^United States Department of Agriculture, Washington, DC, USA

**Keywords:** One Health, competency-based education, interdisciplinary studies, professional competence, medical education, veterinary education

## Abstract

The emergence of complex global challenges at the convergence of human, animal, and environmental health has catalyzed a movement supporting “One Health” approaches. Despite recognition of the importance of One Health approaches to address these complex challenges, little effort has been directed at identifying the seminal knowledge, skills, and attitudes necessary for individuals to successfully contribute to One Health efforts. Between 2008 and 2011, three groups independently embarked on separate initiatives to identify core competencies for professionals involved with One Health approaches. Core competencies were considered critically important for guiding curriculum development and continuing professional education, as they describe the knowledge, skills, and attitudes required to be effective. A workshop was convened in 2012 to synthesize the various strands of work on One Health competencies. Despite having different mandates, participants, and approaches, all of these initiatives identified similar core competency domains: management; communication and informatics; values and ethics; leadership; teams and collaboration; roles and responsibilities; and systems thinking. These core competency domains have been used to develop new continuing professional education programs for One Health professionals and help university curricula prepare new graduates to be able to contribute more effectively to One Health approaches.

## Background and Rationale

The last decade of the twentieth century and the first decade of the twenty-first century saw the emergence of a plethora of public health challenges at the convergence of human, animal, and environmental health, including bovine spongiform encephalopathy (BSE) and variant Creutzfeldt–Jakob Disease, H5N1 influenza, Nipah virus, West Nile Virus, 9/11 and the threat of bioterrorism, SARS, and the impact of climate change on global food systems. While the concept of zoonotic disease (i.e., diseases that are transmitted from animals to humans), and social and environmental determinants of health are well recognized in the public health community, education of medical professionals, by and large, remains segregated between human health (human medicine, nursing, public health), animal health (veterinary medicine, agricultural workers), and environmental health (ecologists).

A 2007 Salzburg Global Seminar, “New Century, New Challenges, New Dilemma: The Global Nexus of Animal and Public Health,” recognized that progress on emerging issues like pandemic influenza response and global food security was slowed by the lack of communication and collaboration among different health professions and the public and private sectors. The seminar focused attention on the need for training health professionals to think globally and facilitate transdisciplinary approaches to health improvement that bring together human, animal, and environmental health practitioners (1). Participants from public, private, academic, and philanthropic organizations reached consensus that this new group of professionals, referred to as “One Health” practitioners, needed to possess unique professional competencies, including “soft skills” to complement the depth of knowledge in their individual areas of expertise, although no specific set of core competencies was developed at that time.

The One Health approach recognizes that the health of humans, animals, and the environment are interdependent and that promoting optimal health in any of these sectors requires cross-sectoral collaboration, communication, and respect (2). The American Veterinary Medical Association defines One Health as “the collaborative effort of multiple disciplines – working locally, nationally, and globally – to attain optimal health for people, animals, and our environment” (3). Implementation of a One Health approach requires a team effort that brings together professionals who come from a variety of disciplines, including human medicine, veterinary medicine, ecosystem health, and agriculture. The Word Health Organization, World Organisation for Animal Health, and the Food and Agriculture Organization of the United Nations have recognized their shared responsibility to use One Health approaches for addressing a number of complex global challenges, such as rabies and antimicrobial resistance (4).

Although the One Health approach has resurfaced in recent years as a strategy to address complex problems at the interface of human, animal, and environmental health, little effort has been directed at identifying the seminal knowledge, skills, and attitudes necessary for individuals to successfully contribute to One Health efforts. The multidisciplinary nature of the One Health approach requires that One Health professionals are proficient in knowledge, skills, behaviors, and attitudes that go beyond the discipline-specific knowledge gained through traditional training programs. Identifying a set of One Health core competencies is critical to prepare professionals to tackle the health threats of the twenty-first century by working collaboratively with peers in other areas of expertise using a One Health approach. Education and training programs, which incorporate these core competencies, will create a workforce better able to address One Health challenges (5, 6).

## The Pedagogical Principles Underlying Competency-Based Education and Training

Traditionally, professional training curricula and academic course design have been guided by topic-specific and knowledge-based standards. A competency-based approach also considers the skills, behaviors, and attitudes that graduates need to become proficient in their future profession (7). Although initially developed as a way to improve corporate strategy (8), core competencies have been found to provide an excellent foundation for developing professionally relevant training programs that better equip individuals to work collaboratively (9). Professionals working in interdisciplinary environments using One Health approaches to address complex public health challenges must possess knowledge, skills, behaviors, and attitudes that go beyond their discipline-specific knowledge. Identifying these core competencies is necessary to develop relevant training programs for One Health professionals (7–10).

The public health profession has recognized the value of core competencies for more than a decade. In 2006, the Association of Schools of Public Health adopted a competency-based educational model for the Master of Public Health degree. The model currently recognizes 12 domains and a total of 119 core competencies (11). In 2010, the Council on Linkages Between Academia and Public Health adopted a set of eight core competency domains to strengthen the public health workforce (12). Within each of these domains, core competencies were delineated for three tiers of public health positions: entry level, program management/supervisory level, and senior management/executive level. The domains in each of these models represent logical content categories that encompass multiple competencies. Domains are critical for curriculum development as they indicate the main areas in which a professional should be competent. Domains are intended to be stable over time, although the individual core competencies within a domain may be adapted for specific course goals (13). Because of their transferability, core competency domains are particularly useful when developing training programs for interdisciplinary fields.

Between 2008 and 2011, three groups independently embarked on separate initiatives to identify core competencies for One Health professionals – the Bellagio Working Group supported by the Rockefeller Foundation and University of Minnesota; the Stone Mountain Meeting (SMM) Training Workgroup; and the RESPOND Initiative funded by the United States Agency for International Development’s Emerging Pandemic Threats Program (USAID/RESPOND). An additional activity, a 2012 workshop in Rome, was designed to integrate the findings of these three groups and synthesize a single set of One Health competencies. Here, we provide a brief overview of each of the original initiatives, a description of the core competency domains they have identified, and some examples of how these core competency domains have been used to develop training programs for One Health professionals.

### Bellagio Working Group

The 2007 Salzburg Seminar set the stage for a follow-up activity sponsored by the Rockefeller Foundation. In September 2008, the University of Minnesota convened a multinational, multidisciplinary workgroup at the Rockefeller Foundation’s Bellagio Center in Bellagio, Italy, to identify core competencies for global food systems leadership (14). Workgroup participants came from five continents and represented a variety of backgrounds, including governments, academia, non-governmental organizations, the private sector, and intergovernmental organizations, including several who had participated in the Salzburg Seminar. During the week-long process, the relevance of these same leadership competencies for all One Health professionals was recognized since global food security and other food systems issues require a One Health approach. Five major domains and a set of personal characteristics were identified for successful One Health leadership. Following the meeting, indicators of competence in each domain were outlined for three workforce levels: foundational, intermediate, and advanced.

### Stone Mountain Meeting Training Workgroup

The SMM Training Workgroup was established following the May 2010 international meeting *Operationalizing “One Health”: A Policy Perspective – Taking Stock and Shaping an Implementation Roadmap* that took place in Stone Mountain, GA, USA (15). The goal of the SMM was to identify clear and concrete actions to move the concept of One Health from vision to implementation. During the meeting, participants deigned a 3- to 5-year vision of One Health encompassing four main areas: culture change, increased visibility, political will and financial support, and optimal coordinated efforts. Seven activities, including training, were identified as critical to attain this vision, and separate workgroups were formed to address these activities. The Training Workgroup comprised of over 50 subject matter experts from U.S. and international government and non-governmental agencies, academic institutions, and other public, animal, and environmental health organizations.

The Training Workgroup was tasked with several activities to build skills and expertise in One Health, including developing a list of essential core competencies for different levels of One Health practitioner. To draft the One Health core competencies, a wide range of existing core competency frameworks were reviewed, including the Association of Schools of Public Health Global Health Competency Model, the Guide to Senior Executive Service Qualifications of the U.S. Office of Personnel Management, the Leadership Core Competencies of the Farm Service Agency of U.S. Department of Agriculture, and the Core Learning Activities for the Nigerian Field Epidemiology and Laboratory Training Program. Through discussions with Workgroup members, the core competencies were refined to be more specific to One Health. The Workgroup identified essential core competency domains for three levels of One Health practitioners – field and entry level workers, program and project managers, and national policy leaders. Each domain was further defined with specific core competencies (16).

### USAID/RESPOND

A multiagency Global One Health Core Competency Workgroup was assembled in 2011 under Tufts University leadership to develop a One Health core competency framework which could be used as a template to assess existing curricula and as an aid in the development or strengthening of university curriculum and workforce training programs (13). The workgroup was funded through USAID/RESPOND with the goal to build capacity for medicine, veterinary medicine, nursing, and public health faculty and trainees from countries in Southeast Asia (Thailand, Indonesia, Vietnam, and Malaysia) and Central and Eastern Africa (Republic of the Congo, Ethiopia, Uganda, Kenya, Tanzania, and Rwanda). Since these regions are “zoonotic hotspots,” or areas where the risk of disease emergence is high, building capacity was critical to enable countries to manage pandemic threats in collaboration with each other and governmental agencies both within a country and across an entire region. Members of the workgroup included representatives of the United States Department of Agriculture, the Centers for Disease Control and Prevention, and academia.

The workgroup met several times in 2011 and 2012 and identified core competencies through a literature review of existing core competency frameworks in the public health, veterinary medicine, and infectious disease fields; telephone interviews with subject matter experts; and iterative feedback from interactive workshops with RESPOND African and Southeast Asian institutional partners in veterinary medicine, public health, nursing, and human medicine. The Workgroup distilled these core competencies into a set of One Health core competency domains and subdomains.

### Rome Synthesis

Through funding provided by the Rockefeller Foundation, the Centers for Disease Control and Prevention, and the Food and Agriculture Organization of the United Nations, the University of Minnesota sponsored a workshop in Rome, Italy, in March 2012 to synthesize One Health Global Core Competencies (17). Attendees came from a broad range of One Health disciplines across the globe, and all worked actively on One Health approaches. Many of the attendees had participated in one or more of the previously mentioned efforts for developing One Health core competency frameworks. The workshop consisted of presentations from each of the different efforts, large group discussions, and small group activities. The immediate goal of the workshop was to validate the existing work related to One Health core competencies. The long range goal was to develop a consensus framework for One Health core competency domains that could be utilized by the global One Health community.

Workshop attendees reviewed nine existing competency frameworks to identify commonalities, including three specific to One Health (the previously mentioned frameworks developed by the Bellagio Working Group, the SMM Training Workgroup, and USAID/RESPOND). Attendees of the Rome meeting found that although the three One Health oriented groups developed their core competencies independently and drew from the expertise of a variety of professional disciplines, each initiative identified similar core competency domains (Table 1). These commonalities among the independent efforts were used to describe seven core competency domains, which were further defined with specific One Health core competencies. The major core competency domains identified were management, communication and informatics, values and ethics, leadership, team and collaboration, roles and responsibilities, and systems thinking.

**Table 1:** **Major One Health core competency domains identified during the Rome synthesis meeting using the Bellagio Working Group, the Stone Mountain Meeting Training Workgroup, and the RESPOND initiative core competency frameworks**.

Source	Major domains													
	Management	Communication and informatics	Values and ethics	Leadership	Team and collaboration	Roles and responsibilities	Systems thinking
Bellagio working group	Working across boundaries	Communication	Ways of being^a^	Visionary and strategic	Influence	Change makers/achieving results	Working across boundaries
SMM	Human capital management, resource management	Communicates lessons learned, communication	Integrity	Vision integration	Collaboration; diplomacy; builds diverse teams; interpersonal skills	Problem solving; flexibility; self-development	External awareness; strategic thinking
USAID/RESPOND	Planning and management; analysis and assessment	Applied informatics; communication, and collaboration	Ethics and professionalism	Leadership and systems thinking	Communication and collaboration	Leadership	Leadership and systems thinking; cultural competence; policy and regulation
Rome synthesis	Leadership and management	Communication	Values and ethics	Leadership and management, conflict resolution	Teamwork		Systems analysis/thinking (external awareness and big picture); creating an enabling environment and advocating change
Example competencies proposed during the Rome synthesis	Able to manage cross disciplinary teams – understands roles and responsibilities of team and its individual members – holds team accountable	Utilizes diplomacy – able to negotiate – able to resolve conflicts – achieves collaboration	Values honesty – possesses strong knowledge of self – possesses integrity	Advocates for change – fosters a change environment – understands individual and shared leadership models – possesses an external awareness (social, political, legal, and cultural)	Able to identify shared values and goals – values diversity of discipline, culture, ideas, background, and experience – establishes trust – thinks strategically		Awareness of big picture and interdependency of stakeholders – understands and embraces a One Health approach – able to identify problem and its impact on the system

*^a^Used to describe a portfolio of value-driven personal attributes: confidence, courage, credibility, emotional intelligence, empathy, ethics, passion, self-awareness, spirituality, and wisdom*.

## Results to Date

These One Health core competency frameworks outline the skills and behaviors necessary for successful performance within the One Health workforce. Educators at all levels of One Health training can use these frameworks as a resource for curriculum development. The competency framework is flexible enough to allow educators the opportunity to tailor their learning objectives to local needs.

The Bellagio model has been used to develop in-service One Health leadership training programs in Africa, Asia, and the Americas aimed at university teaching staff and government officials. In 2010, Chiang Mai University, Thailand, piloted a One Health Leadership continuing professional education course based on the Bellagio model. The course is now the cornerstone of a Global Health Institute held annually at Chiang Mai University. These continuing professional education courses provide intensive experiential learning for working professionals over the course of 2–5 days. Self-assessment, individual skill building, group exercises, and reflections are used to create a positive climate for behavior change among adult learners.

Tufts University, along with USAID/RESPOND partners at the University of Minnesota Colleges of Veterinary Medicine, Public Health, Nursing, and Medicine, is using their framework with institutional partners across Southeast Asia and Africa. Having faculty from multiple disciplines, institutions, and countries agree on the major competency domains meant that the competency framework could be used to develop specific training modules to address the individual domains. The competency framework has been used to evaluate coverage in existing curricula, enhance current courses, and create new ones. Graduate programs emphasizing One Health approaches use the framework to guide curriculum design. Finally, the framework also facilitates identification of faculty development needs.

Faculty have used these frameworks to improve One Health programs and training for graduate students and early career professionals at the University of Minnesota. For example, faculty from the College of Veterinary Medicine, School of Public Health, and Humphrey School of Public Affairs created a new course, “Leadership to Address Global Grand Challenges,” that is built around the One Health competency domains described here. Originally listed as a public health course entitled “Global One Health Leadership,” the early course enrollment was primarily Master of Public Health Students. Changing the name to “Leadership to Address Global Grand Challenges” and listing the course in public affairs rather than in public health increased the diversity of students registering to include graduate students from veterinary medicine, public health, agriculture, liberal arts, education, and public affairs. The learning objectives of this course demonstrate the integration of the One Health competencies (Table 2). The course differs from most other graduate courses in that skill building and group activities predominate instead of lectures. The course also involves student-facilitated discussions with internal and external stakeholders on current complex challenges. Feedback from the course is overwhelmingly positive, with participants commonly citing the immediate usefulness of these skills in their jobs, communities, and families. One hundred percent (100%) of the 28 participants in the last three One Health leadership courses taught in Thailand and the United States have mentioned communication competencies in their reflections on the question “What new One Health skills have I learned that I can use immediately?” The specific communications skills most commonly cited were listening (61%), collaboration skills (46%), and appreciating differences (29%).

**Table 2:** **Course objectives for PA5152, “Leadership to Address Global Grand Challenges: Focus on Food Systems,” held May 23–27, 2016, at the University of Minnesota**.

Course objectives
At the conclusion of the course, participants will have a deeper understanding of – and an opportunity to apply – leadership skills that foster collective action across industry, government, academia, and civil society sectors. Specifically, participants will:
1. Expand meta-leadership skills for:
a. Leading one’s self b. Leading in teams (where you have authority and responsibility) c. Leading up in organizations (where you may have responsibility but no authority) d. Leading across (where you have neither responsibility or authority)
2. Deepen understanding of global food system grand challenges and why they require integrative leadership approaches
3. Learn about, observe, and practice specific integrative leadership skills that include:
a. Listening to understand and be understood b. Building trust and credibility c. Facilitating dialog, debate, and deliberation d. Aligning policy implementation tools with desired outcomes e. Anticipating and leveraging windows of opportunity f. Designing inclusive structures and facilitating decision-making processes g. Providing constructive feedback and fostering continuous self-improvement

In addition to providing guidance for curriculum and training design, core competency frameworks can be used to evaluate existing training programs. The SMM Training Workgroup plans to use their list of core competencies to identify gaps in existing One Health training programs. For example, involvement in the development of the One Health core competencies helped the One Health Coordination Center in the Animal and Plant Health Inspection Service of the United States Department of Agriculture identify the need for new in-service training of field staff so that staff members were better able to foster collaboration with local public health agencies. The One Health Systems Mapping and Analysis Resource Toolkit (OH-SMART) was designed to strengthen systems thinking competencies.

## Discussion

Although the three groups described here developed their core competencies independently and employed expertise from different disciplines, each initiative identified similar core competency domains. This consistency further substantiates the validity of these domains for One Health professionals. None of these domains are specific to any single discipline and, thus, can be easily adapted by different professions and institutions across the globe. Although this paper focused on the core competency domains, the individual core competencies within these domains can provide additional guidance for developing curricula relevant to local and national One Health needs.

These One Health core competency frameworks provide a common foundation for continuing professional education and training programs that move beyond the focus on discipline-specific knowledge. The overarching goal of One Health training is to impart trainees with the non-technical skills and attitudes that all One Health practitioners need, regardless of discipline or work level. Although many of the domains identified in this review are not traditionally incorporated into discipline-specific training programs, faculty who work in the field of One Health feel that such training is long overdue. The impetus driving the development of these One Health competency domains reinforces the need to equip public health professionals with a breadth of skills to complement their own specific areas of expertise.

The core competency frameworks designed by USAID/RESPOND and the Bellagio Working Group, and the core competency synthesis from the Rome Synthesis meeting were designed to assist in the development of One Health continuing professional education. These frameworks can be used by universities, governments, and regional networks as a starting point for identifying specific core competencies relevant to local needs, which can in turn be used to guide development of new training programs. Educators also can use the domains to identify strengths within existing curricula, as well as opportunities in the curricula to incorporate additional One Health learning needs.

Curriculum change is difficult, in part because acknowledging a need to change suggests to some that the current curriculum and/or the instructors delivering it are inadequate. Agreement on a set of competency domains may be less threatening as it acknowledges that the specific competencies in each domain vary by setting and require constant tweaking to meet the needs of the changing world around us. Some competencies can be achieved by modifying existing courses rather than creating brand new ones. For example, USAID/RESPOND is aware that many programs are overcrowded with material and encourages integrating training modules into existing courses, rather than trying to create new zoonotic disease and pandemic management courses. USAID/RESPOND worked with faculty to think about where existing cases could be replaced with cases designed to highlight One Health competencies.

The Global Health Security Agenda (GHSA) has recognized the importance of multidisciplinary coordination to prevent, detect, and respond to the disease threats facing health systems around the globe (18). In addition to an overall recommendation of utilizing multidisciplinary coordination to meet GHSA targets when possible, the GHSA Workforce Development Action Package specifically recommends the development of a multi-sectoral workforce to better detect global health threats (19). The One Health competency domains can serve as a resource for countries implementing the GHSA Workforce Development Action Package to ensure that graduates of their training programs are equipped to handle the complex health threats in their countries and regions.

As transdisciplinary One Health approaches continue to gain more attention worldwide, these common core competency domains can provide a foundation for developing and revising One Health training programs. Educators and organizations wishing to adopt One Health approaches can use these domains as a framework to help them evaluate current curricula, identify gaps and opportunities for enhancement of design curricula, and develop evaluation instruments.

## Author Contributions

CR and RF coordinated all workgroup activities associated with the Stone Mountain Meeting. KC and JA led the Stone Mountain Meeting Training Workgroup and the development of the workgroup’s core competency domains. ML and RH led development of the USAID One Health Core Competency Framework. WH and LV led the Bellagio Working Group. WH and DO led the Rome Synthesis. WH and LV developed new university courses and continuing education programs based on these OH competencies. RF drafted the initial manuscript draft. All authors contributed to the idea and manuscript framework, revisions, and final manuscript approval.

## Conflict of Interest Statement

The authors declare that the research was conducted in the absence of any commercial or financial relationships that could be construed as a potential conflict of interest.
